# Attitudes and Recommendations of Physicians towards Alcohol Consumption and Cardiovascular Health: A Perspective from Argentina

**DOI:** 10.3390/diseases6030077

**Published:** 2018-09-01

**Authors:** Ricardo Lopez Santi, Sohaib Haseeb, Bryce Alexander, Adrian D′Ovidio, Sergio Gimenez, Carlos Secotaro, Diego Martinez Demaria, Luis Maria Pupi, Sonia Costantini, Daniel Piskorz, Alejandro Amarilla, Alberto Lorenzatti, Narcisa Gutierrez, Wilma Hopman, Adrian Baranchuk

**Affiliations:** 1 Division of Cardiology, Hospital Italiano de La Plata, Buenos Aires B1900, Argentina; lopezsan@live.com.ar (R.L.S); diegorodrigo58@yahoo.com.ar (D.M.D.); 2 Department of Medicine, Queen’s University, Kingston, Ontario K7L 2V7, Canada; sohaib.haseeb@queensu.ca (S.H.); balexander@qmed.ca (B.A.); Wilma.Hopman@kingstonhsc.ca (W.H.); 3 Division of Cardiology, Hospital Rawson, San Juan J5402DRJ, Argentina; ahdovidio@gmail.com; 4 OSEP, Obra Social de los Empleados Públicos, Mendoza M5500, Argentina; sergiogimenez16@hotmail.com; 5 Division of Cardiology, CMP, Penta Medicina Cardiovascular M5500, Argentina; secotaro@gmail.com; 6 Division of Cardiology, Clínica del Sol, Ciudad Autónoma de Buenos Aires C1425AYM, Argentina; lmpupi52@gmail.com; 7 Especialistas en Cardiología SRL, General Roca, Rio Negro R8332, Argentina; sscostantini@yahoo.es; 8 Cardiovascular Research Center of Sanatorio Británico SA, Rosario, Santa Fe S2000, Argentina; daniel.piskorz@hotmail.com; 9 Institute of Cardiology Juana F. Cabral, Corrientes W3400CDS, Argentina; alexamarilla@hotmail.com; 10 Medical Institute/Rusculleda Foundation for Research, Cordoba X5022, Argentina; alberto.lorenzatti@gmail.com; 11 Department of Cardiology, Instituto Médico de Alta Complejidad IMAC, Salta A4400, Argentina; narcisa.gutierrez@gmail.com

**Keywords:** alcohol drinking, health knowledge, physician attitudes, standard drink, wine

## Abstract

Despite epidemiological findings of improvements in cardiovascular risk factors with a light-to-moderate intake of alcohol, many misconceptions remain regarding alcohol intake and the risks and benefits of consumption. We sought to examine physician attitudes and recommendations regarding alcohol intake in a cohort of Argentine physicians and to establish their sources of knowledge. An online national survey was distributed through the Argentine Federation of Cardiology (FAC) to cardiologists, internal medicine specialists, general and other subspecialty physicians in Argentina. The survey was completed by 745 physicians, of whom 671 (90%) were cardiologists. In total, 35% of physicians viewed moderate alcohol intake to be beneficial for cardiovascular health, 36% believed only wine offered such benefits, 24% viewed any intake to be harmful, and 5% had other opinions. More than half (57%) self-reported their knowledge came from academic sources. Regarding knowledge of drinking guidelines, only 41% of physicians were aware of the concept of “standard drink”. Physicians were generally not comfortable converting standard drinks into other metric units, however men tended to be more comfortable than women (*p* = 0.052). Physicians were not satisfied with their knowledge of drinking guidelines (3.01 ± 2.73, on a 0–10 scale). Physicians were generally comfortable in counselling patients regarding safe limits of consumption (6.22 ± 3.20, on a 0–10 scale). Argentine physicians were not satisfied with their knowledge of alcohol consumption guidelines or their understanding of the reported metrics. Only one-third of study participants viewed moderate alcohol intake as beneficial for cardiovascular health. This study shows the necessity to optimize the sources of knowledge.

## 1. Introduction

Alcoholic beverages have been consumed for thousands of years [1,2,3]. In 2017, global wine consumption was 243 million hectoliters (mhl), with Argentina being the eighth largest global consumer of wine (8.9 mhl) [4]. Argentina is also the sixth largest global producer of wine (11.8 mhl of 250 mhl) [4]. Despite the wide popularity, excessive consumption of wine and alcohol is a major risk factor for morbidity and mortality; alcohol contributes to 4% of all deaths and plays a putative role in 60 different diseases including atrial fibrillation, hypertension, and cirrhosis [5,6,7]. Alcohol is connected to more than 200 International Classification of Disease (ICD-10) codes and its chronic heavy misuse has substantially contributed to the global burden of disease [8,9,10]. The non-heavy use of alcohol, at light-to-moderate amounts, has been linked to a reduction in the risk of multiple cardiovascular outcomes [11,12,13]. Despite many multicenter and cross-cultural epidemiological studies in agreement, there is conflicting evidence, and in the absence of controlled clinical trials, the causal nature of this observation continues to be a heavily debated topic in the medical and lay literature [14,15,16,17]. Healthcare providers are well positioned to counsel patients regarding appropriate levels of alcohol intake and its risks and benefits of consumption. This study sought to investigate physicians’ attitudes and recommendations towards alcohol intake in a cohort of Argentine physicians and to establish their sources of knowledge.

## 2. Materials and Methods

### 2.1. Study Population

The study population included practicing cardiologists, internal medicine specialists, general and other specialty physicians residing in Argentina; trainees were eligible. Academic and non-academic physicians were identified through the Argentine Federation of Cardiology (FAC) mailing list, which endorsed this study. The study was approved by the Research Ethics Board of Queen’s University (file # 6022829).

### 2.2. Study Design

A cross-sectional online survey consisting of 25 questions was developed using Google Forms (Mountain View, CA, USA). Questions examined participant demographics, perceptions on alcohol and cardiovascular health, and knowledge and attitudes towards drinking guidelines. Questions contained dichotomous, Likert-type, rank-order, and open-ended response choices. Questions were not forced and respondents were permitted to select multiple response choices depending on the question content. The survey was developed using topics and themes identified in updated literature reviews [1,2,3,5,6,7] and with input from the study investigators.

### 2.3. Study Distribution

The survey was distributed across the 7 predetermined geographic regions of Argentina via the Argentine Federation of Cardiology, the FAC (Supplementary Table S1). Potential participants from each region were sent unique, but not individualized, links to the survey by appointed regional champions; members of the FAC, as well as other practicing physicians in the regions, were invited. The regional champions were responsible for tracking the number of survey recipients to determine the national and regional response rates. The survey was distributed between 1 February 2018 and 3 June 2018 to a non-overlapping cohort of physicians. Reminders were periodically emailed to maximize the response rate, with no incentives.

### 2.4. Statistical Analysis

Data were initially collected in Google Forms and exported into IBM SPSS (version 24.0 for Windows, Armonk, NY, USA) for statistical analysis. Data were described using means and standard deviations for continuous variables, and frequencies and percentages for categorical variables. Independent sample T-tests were used to compare the normally distributed continuous variables, the Mann-Whitney U was used for non-normally distributed continuous variables, and the Pearson chi-squared test (or the Fisher’s Exact test as appropriate) for categorical variables. A *p* value of less than 0.05 was considered statistically significant, and no adjustments were made for multiple comparisons.

## 3. Results

### 3.1. Population Demographics

Survey invitations were distributed to 1334 physicians. There were 745 total respondents, of which 671 were cardiologists, 18 internal medicine specialists, 17 general physicians, 36 other specialty physicians, and 3 resident trainees. The overall response rate for the survey was 56%. The regional response rates and other geographic characteristics are summarized in Supplementary Table S1. The demographic and clinical practice characteristics are summarized in Table 1. Most respondents were male (71%; 524/745); 63% (467/745) of physicians practiced in regions that were designated as non-producers of wine, and 37% (278/745) of physicians practiced in regions that were producers of wine. The majority of physicians (72%; 535/745) practiced in non-academic centers.

### 3.2. Perceptions on Alcohol and Cardiovascular Health

**Figure 1:**
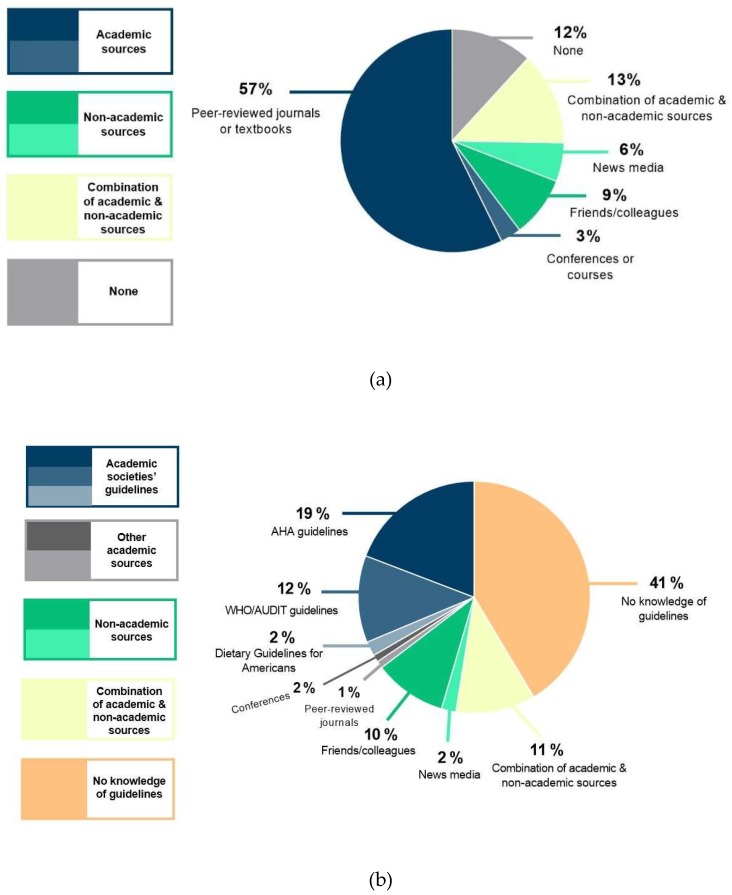
Physicians’ self-reported sources of knowledge on (**a**) alcohol and cardiovascular health, and (**b**) alcohol consumption guidelines.

Regarding physicians’ perceptions on alcohol, 35% (257/737) viewed moderate alcohol intake to be beneficial for cardiovascular health, 36% (264/737) believed only wine offered such benefits, 24% (179/737) viewed any intake to be harmful, and 5% (37/737) had other opinions. To a healthy patient, approximately two-thirds (64%; 475/739) of physicians would be comfortable recommending a light-to-moderate pattern of consumption, given the patient is a drinker. To a patient at risk of heart disease who drinks alcohol, less than half (46%; 343/743) of physicians would recommend cessation of alcohol, however more than half (54%; 400/743) would be comfortable with the patient consuming alcohol; of those, the majority (86%; 343/400) of physicians would recommend a light-to-moderate pattern of consumption. More than half (57%; 439/728) of physicians self-reported their knowledge came from academic sources (Figure 1A). The type of institution, gender, or clinical practice setting did not significantly affect physicians’ perceptions.

### 3.3. Knowledge of Drinking Guidelines

**Figure 2:**
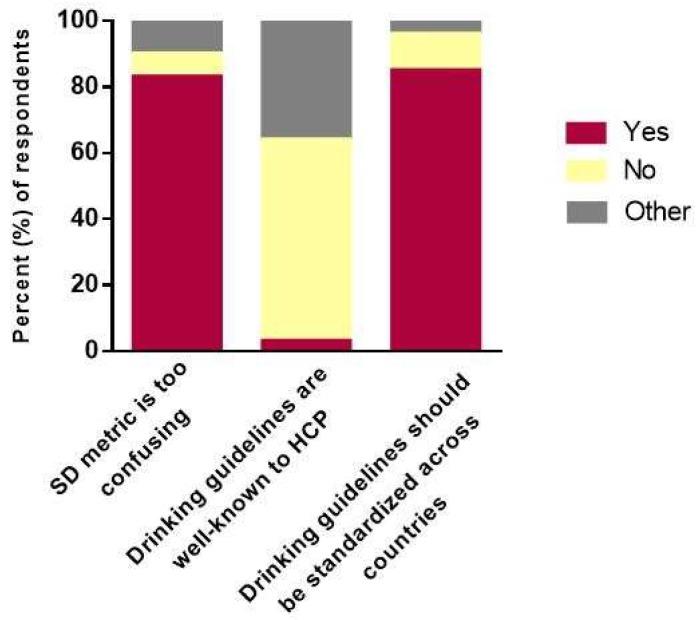
Physicians’ attitudes on the current state of drinking guidelines. Abbreviations: HCP = healthcare providers; SD = standard drink.

As shown in Table 2, physicians were not satisfied with their current knowledge and understanding of the drinking metrics and guidelines. Male physicians were significantly more satisfied than female physicians (3.25 ± 2.73 vs. 2.47 ± 2.5; *p* < 0.001). Only 41% (301/739) of physicians were aware of the concept of “standard drink”. Physicians were not comfortable converting standard drinks into other common metric units (1.78 ± 2.52), however male physicians tended to be more comfortable than female physicians (1.90 ± 2.57 vs. 1.50 ± 2.38; *p* = 0.052). The overwhelming majority (83%) found this metric to be confusing, believed that drinking guidelines were not well-known to healthcare providers (61%), and preferred the drinking guidelines to be standardized across countries (85%) (Figure 2). Despite this, physicians were generally comfortable in counselling patients regarding the safe-limits of consumption (6.22 ± 3.20), with a trend towards male physicians being more comfortable than female physicians (*p* = 0.071) (Table 2). Physicians’ self-reported sources of knowledge for drinking guidelines and suggestions for future educational strategies are summarized in Figure 1B and Table 3, respectively.

## 4. Discussion

The results of this study show that Argentine physicians had variable perceptions of alcohol as they relate to cardiovascular health. They were not satisfied with their current knowledge of drinking guidelines, or understanding of the reported metrics. Previous studies have shown that healthcare professionals use a variety of screening and intervention methods to aid high-risk alcohol users [18]. A survey of German primary care physicians regarding lifestyle-based prevention for cardiovascular diseases showed that only a fraction of the physicians provided routine intervention for alcohol consumption [19]. A 2001 Canadian survey of obstetricians, midwives, and family physicians on alcohol and pregnancy found that the providers’ definition of “moderation” varied, which influenced their medical advice [20]. Despite the aforementioned studies, there is little direct evidence on physicians’ perceptions on alcohol and cardiovascular health, and their knowledge and attitudes towards drinking guidelines. With an increased awareness on the perils of alcohol misuse, drinking guidelines have been adopted in at least 37 countries to promote safe drinking practices [21]. Guidelines on “low-risk” drinking are usually based on meta-analyses and quantitative overviews of observational studies [22,23], however, it is quite apparent from the literature that there are cross-national variations in maximum consumption limits, standard drink sizes, and low-risk thresholds [24]. A study by Wood et al. [25] improved on previous meta-analyses to define the low-risk drinking thresholds associated with all-cause mortality and cardiovascular disease. Analysis of individual-participant data for alcohol use from 83 prospective studies in 19 high-income countries found that the lowest risk of all-cause mortality was at levels of 100 g/week or less. This threshold was supported by the UK’s drinking guidelines [26], but was substantially lower than the guidelines instituted in many high-income countries, particularly in the United States [27].

Drinking guidelines are usually reported in standard drinks [28]. The World Health Organization’s (WHO) Alcohol Use Disorders Identification Test (AUDIT) defines 1 standard drink (SD) as equal to 10 grams of pure ethanol [29]. The WHO’s low-risk drinking guideline for current drinkers is ≤2 SD per day with at least 2 non-drinking days per week. The US Dietary Guidelines [27] define 1 SD as equal to 14 grams of pure ethanol, with a low-risk guideline of ≤2 SD per day for men and ≤1 SD per day for women. The UK’s drinking guidelines define 1 SD as equal to 8g of pure ethanol, with a low-risk guideline of 14 SD per week spread evenly over 3 days or more [30]. The American Heart Association [31,32], American Stroke Association [33], American Society of Hypertension [34], and the American Diabetes Association [35] have devised their own guidelines. In our cohort of highly trained cardiologists, these discrepancies between institutional guidelines can be a contributing reason to their underwhelming knowledge.

There are limitations to the present study that warrant mention. The study employed a survey-based design where the findings are based exclusively on self-reported data; although practical, the extent to which the self-report translates to clinical practice cannot be discerned. There is a potential for selection bias because physicians who consume alcohol might be more likely to respond. Although the study had national representation and a high response rate, respondents were predominantly cardiologists who practiced in Argentina. As such, the findings may not be representative of other healthcare providers or generalizable across countries.

## 5. Conclusions

Argentine physicians displayed variability in their perceptions of alcohol and its effects on cardiovascular health. They were not satisfied with their current knowledge of drinking guidelines, or understanding of the reported metrics. The identification of these knowledge gaps at a national level provides a critical starting point for further investigations and show the necessity to optimize the sources of knowledge.

## Figures and Tables

**Table 1:** Demographic and clinical practice characteristics stratified by regional location of the physicians’ clinical practice.

Variable	All Respondents ^1^ (*n* = 745)	Respondents Location ^1^		*p*-Value
		Non-Producer Regions (*n* = 467)	Producer Regions (*n* = 278)	
Age, *n* (%)				0.068
<35 years	116 (16)	63 (14)	53 (19)	
35–44 years	199 (27)	116 (25)	83 (30)	
45–54 years	202 (27)	134 (29)	68 (25)	
55–64 years	145 (20)	100 (22)	45 (16)	
>65 years	72 (10)	46 (10)	26 (10)	
Gender, *n* (%)				0.492
Male	524 (71)	324 (70)	200 (72)	
Female	217 (29)	140 (30)	77 (28)	
Clinical practice setting, *n* (%)				
Urban or rural				0.721
Urban	712 (95)	444 (95)	268 (96)	
Rural	2 (1)	1 (1)	1 (1)	
Both	29 (4)	20 (4)	9 (3)	
Academic or non-academic				<0.001
University hospital (A)	194 (26)	142 (31)	52 (19)	
Private academic (A)	12 (2)	9 (2)	3 (1)	
Private hospital (NA)	238 (32)	155 (33)	83 (30)	
Private clinic (NA)	224 (30)	123 (27)	101 (37)	
Community hospital (NA)	63 (9)	29 (6)	34 (12)	
Other (NA)	10 (1)	6 (1)	4 (1)	

^1^ Due to the non-forced nature of the survey, respondents were permitted to leave questions blank; cell counts may not always equal the sample size because of small amounts of missing data for age (*n* = 11), gender (*n* = 4), urban or rural practice (*n* = 2), academic or non-academic practice (*n* = 4).

**Table 2:** Physicians’ self-reported knowledge and understanding of drinking metrics and guidelines.

Variable	All Respondents ^1^ (*n* = 745)	Gender ^1^		*p*-Value
		Male (*n* = 524)	Female (*n* = 217)	
Knowledge of drinking guidelines				
Satisfaction with own knowledge, mean ± SD ^2^	3.01 ± 2.73	3.25 ± 2.73	2.47 ± 2.50	<0.001
Satisfaction in guiding patients, mean ± SD ^2^	6.22 ± 3.20	6.39 ± 3.11	5.92 ± 3.34	0.071
Knowledge of drinking metric units				
Aware of metric “standard drink”, *n* (%)				0.099
Yes	301 (41)	217 (42)	83 (39)	
No	351 (47)	250 (48)	98 (45)	
Maybe	87 (12)	53 (10)	34 (16)	
Satisfaction with converting standard drinks to other metrics, mean ± SD ^2^	1.78 ± 2.52	1.90 ± 2.57	1.50 ± 2.38	0.052

^1^ Due to the non-forced nature of the survey, respondents were permitted to leave questions blank; cell counts may not always equal the sample size because of small amounts of missing data for aware of metric “standard drink” (*n* = 6), gender (*n* = 4). ^2^ Likert scale: 0 = not satisfied to 10 = extremely satisfied.

**Table 3:** Most commonly suggested strategies to educate healthcare providers on drinking guidelines.

	Respondents, *n*
Lectures and conferences	426
Website	349
Interactive smartphone app	322
Media campaigns and workshops	3
No need for strategies	18

Note: Respondents could select multiple response choices or suggest other strategies as free-text. For those who selected multiple response choices, a value was assigned to each of the selected choice.

## Supplementary Materials

The following are available online. Table S1: Characteristics of study participants stratified by regions.
