# Supplementary material for: Attitudes and Recommendations of Physicians towards Alcohol Consumption and Cardiovascular Health: A Perspective from Argentina
Source: Diseases. 2018 Sep 1;6(3):77. doi: 10.3390/diseases6030077 (PMC6165240; doi:10.3390/diseases6030077)
Supplement: Supplementary file 1 [file diseases-06-00077-s001.pdf]

Supplementary Appendix

Supplementary Table S1. Characteristics of study participants stratified by regions.

Region #	Region name	Encompassing cities or provinces	Wine producers or non-producers	Denominator (n)	Total responses (n)	Regional response rate (%)	Percentage of total (%)
1	Bonaerense	Buenos Aires, La Plata	NP	156	114	73%	15%
2	Litoral	Rosario	NP	374	196	52%	26%
3	Centro	Cordoba	NP	143	58	41%	8%
4	Sociedades del NEA	Misiones, Formosa, Chaco, Corrientes	NP	210	99	47%	13%
5	Sociedades del NOA	Salta, Jujuy, Tucuman, Santiago del Estero	P	248	115	46%	15%
6	Patagonica	Tierra del Fuego, Neuquen, Comahue	P	53	41	77%	6%
7	Cuyo	San Luis, San Juan, Mendoza	P	150	122	81%	17%

Abbreviations: NP = non-producer; P = producer
